# Time-resolved small-angle neutron scattering as a probe for the dynamics of lipid exchange between human lipoproteins and naturally derived membranes

**DOI:** 10.1038/s41598-019-43713-6

**Published:** 2019-05-20

**Authors:** Selma Maric, Tania Kjellerup Lind, Manfred Roman Raida, Eva Bengtsson, Gunilla Nordin Fredrikson, Sarah Rogers, Martine Moulin, Michael Haertlein, V. Trevor Forsyth, Markus R. Wenk, Thomas Günther Pomorski, Thomas Arnebrant, Reidar Lund, Marité Cárdenas

**Affiliations:** 1 Dept. of Biomedical Science, Malmö University, Per Albin Hanssons väg 35, 205 02 Malmö, Sweden; 2 Singapore Lipidomics Incubator (SLING), Life Sciences Institute, National University of Singapore, Singapore, Singapore; 3 Dept. of Clinical Sciences, Lund University, Jan Waldenströms gata 35, CRC, Box 50332, 212 13 Malmö, Sweden; 4 ISIS Science and Technology Facilities Council, Harwell Science and Innovation Campus, Chilton, Didcot, Oxfordshire OX11 0QX United Kingdom; 5 Life Science Group, Institut Laue Langevin, 6, rue Jules Horowitz, BP 156, F-38042 Grenoble, Cedex 9 France; 6 Faculty of Natural Science and Institute for Science and Technology in Medicine, Keele University, Staffordshire, ST5 5BG United Kingdom; 7 Dept. of Plant and Environmental Sciences, University of Copenhagen, Thorvaldsensvej 40, 1871 Frederiksberg C, Denmark; 8 Dept. of Molecular Biochemistry, Ruhr University Bochum, Faculty of Chemistry and Biochemistry, 44780 Bochum, Germany; 9 Dept. of Chemistry, University of Oslo, Blindern, 0315 Oslo, Norway

**Keywords:** Biophysical chemistry, SAXS

## Abstract

Atherosclerosis is the main killer in the western world. Today’s clinical markers include the total level of cholesterol and high-/low-density lipoproteins, which often fails to accurately predict the disease. The relationship between the lipid exchange capacity and lipoprotein structure should explain the extent by which they release or accept lipid cargo and should relate to the risk for developing atherosclerosis. Here, small-angle neutron scattering and tailored deuteration have been used to follow the molecular lipid exchange between human lipoprotein particles and cellular membrane mimics made of natural, “neutron invisible” phosphatidylcholines. We show that lipid exchange occurs via two different processes that include lipid transfer via collision and upon direct particle tethering to the membrane, and that high-density lipoprotein excels at exchanging the human-like unsaturated phosphatidylcholine. By mapping the specific lipid content and level of glycation/oxidation, the mode of action of specific lipoproteins can now be deciphered. This information can prove important for the development of improved diagnostic tools and in the treatment of atherosclerosis.

## Introduction

Atherosclerosis is a disease in which plaques of lipids and fibrous elements accumulate in the blood vessels^1^. This can lead to the hardening of arteries and eventually to heart disease and stroke^1^. The clinical consequences of atherosclerosis constitute the leading cause of death in the west and double the amount of cancer related death^2,3^. Certain plasma lipoprotein particles such as the high-density lipoprotein (HDL) and low-density lipoprotein (LDL) are associated with the disease and are therefore currently used as clinical markers^4^. Although we count today with ‘lifestyle risk factors’ besides these clinical markers, unexpected deaths on grounds of myocardial and cerebral infarction and stroke are regularly reported even for people who would not normally be considered as high-risk patients^5^. The function of the HDL and LDL particles and their lipid exchange with cellular membranes need to be carefully studied in order to understand the role that these particles play in the buildup of arterial plaques at a microscopic and molecular level.

**Figure 1:**
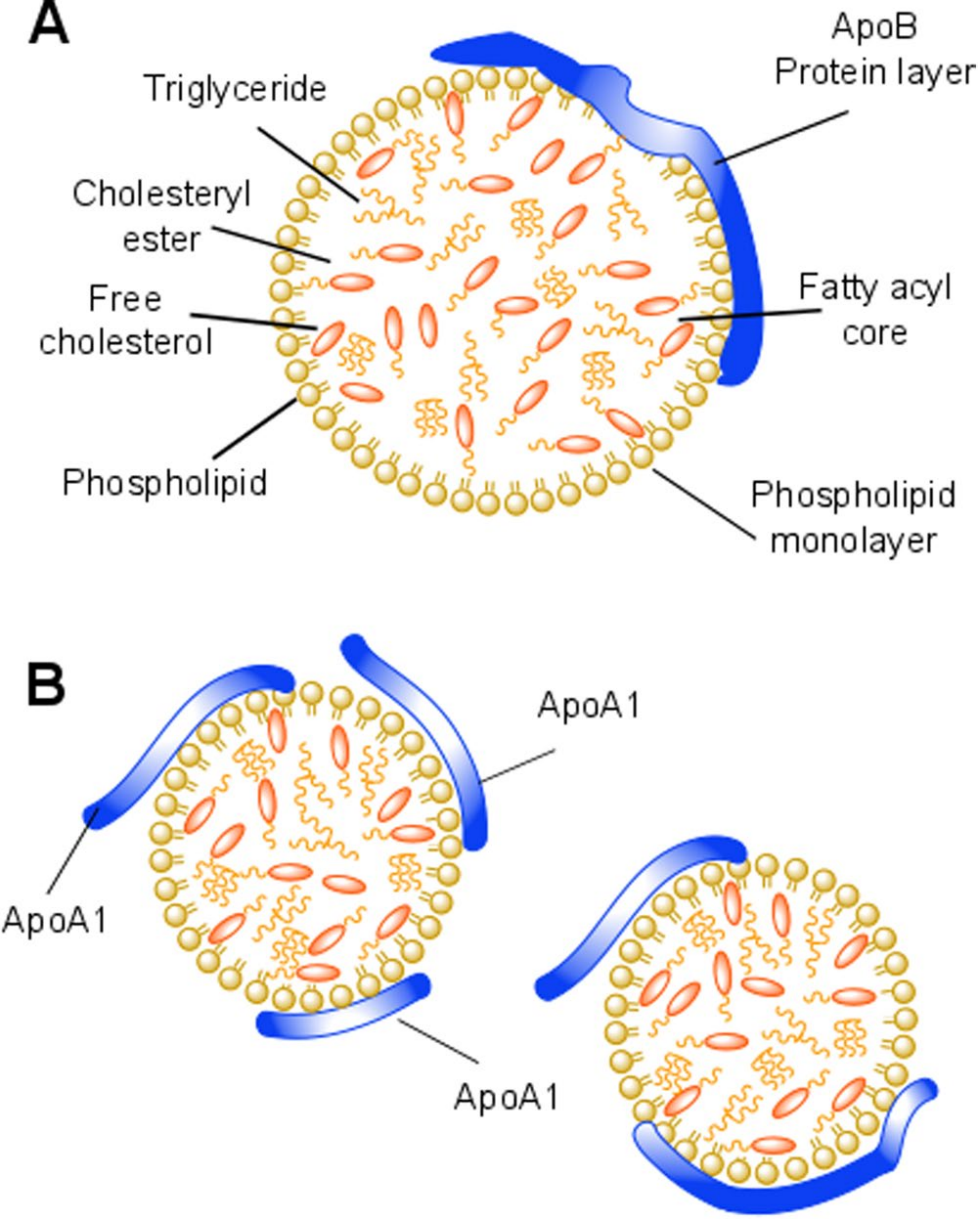
Schematic representation of lipoproteins. (**A**) Illustration of the proposed LDL structure at 37 °C with an inner core consisting of free cholesterol, cholesteryl esters and triglycerides, surrounded by a monolayer of phospholipid fatty acyl tails and an outer hydrophilic layer consisting of the lipid headgroups and apolipoprotein ApoB-100. (**B**) The proposed structural model of HDL with a core structure similar to that of the LDL but including a variable number of apolipoprotein ApoA1.

The lipoprotein particles comprise a heterogeneous group of water-soluble aggregates^6^ (see Fig. 1. for a schematic representation). They are extremely complex in terms of composition, coming in a variety of sizes that range from 5 to 80 nm, and are classified according to their density - the higher the protein content, the higher their density. In addition to the differences in the protein amount, they also differ in protein type as well as the lipid/cholesterol composition^6^. LDL, known as the “bad cholesterol”, accumulates at the interior of the artery wall^7^, where it is oxidized and taken up by foam cells in a process that leads to the development and onset of atherosclerosis^7,8^. HDL, on the other hand, is thought to remove cholesterol from foam cells, limit the inflammatory processes and inhibit the oxidation of LDL, thus disrupting atherogenesis at several key stages^8,9^. This is the basis for the so-called HDL therapy in which artificial HDL particles are thought to remodel the plaque composition. However, a very recent study has shown that an increased level of HDL, associated with a loss-of-function mutation in the HDL receptor SR-B1, can also lead to plaque build-up and increase the risk of cardiovascular disease^10^. The impact of this mutation on HDL metabolism and atherosclerosis risk in humans is however still unclear^10^. Some evidence exist for a decrease in reversed cholesterol transport from foam cells despite high HDL-cholesterol levels^11^.

The lipid composition differs widely between biological membranes. For example, a significant increase in sphingomyelin (SM) content in relation to phosphatidylcholine (PC) is seen in the aorta and the artery wall of humans during aging. This becomes even more pronounced in atherosclerosis^12^. In addition, the quantity of lipid in the intima of the human aorta increases with the degree of atherosclerotic lesion. In the early stages of aortic lipid accumulation, lipids accumulate intracellularly, whereas later on, when fatty streaks and plaques have formed, lipids are mainly present extracellularly^13^. The main fat components in atherosclerotic plaques are cholesterol, cholesteryl esters and phospholipids. Here, the relative ratio of cholesterol esters to phospholipid also increases with the degree of the lesion^13^.

Both lipoproteins and cellular membranes are dynamic lipid-protein structures and as such readily interchange molecules. The movement of lipids between lipid-protein complexes can either occur through exchange, net transfer, or a combination of the two. Understanding the uptake and exchange of lipids by different lipoproteins has been in a research focus for decades and lipid exchange phenomena have been measured both *in vitro* and *in vivo*^14,15^. These studies include results relating to the transfer of phospholipids^14–17^, cholesterol^18–21^ and triglycerides^22,23^. However most of these studies report changes in particle composition upon equlibrium and between lipoparticle types. Only a few studies have followed exchange events that occur between lipoproteins and cells^17^, and lipoproteins and simplified cell models such as lipid vesicles^24^, and lipid microemulsions^25,26^. The complexity of these systems, has left this work inconclusive, and on ocassions contradictory. To resolve this, it is necessary to map the molecular mechanisms underlying plaque buildup, and in particular to characterize the changes taking place both in the lipoprotein particle structure and in the structure of the endothelial cell membranes lining the artery wall during their interplay. Neutron reflectometry has previously been used to gather information on the structure and composition of the membrane itself after interactions with human lipoprotein particles. These studies show that LDL and HDL have differing interaction modes when tested with synthetic, saturated membranes^27,28^. Interestingly, the presence of negatively charged lipids in the synthetic membranes enhanced the uptake of lipids by both lipoproteins, whereas the lipid deposition remained the same^28^. Although the reflectometry studies were able to reveal important information on how the specific membrane composition affects the uptake and deposition of lipids by lipoproteins, this approach did not yield information on how this lipid exchange affects the structure of the lipoproteins.

Small-angle X-ray and neutron scattering (SAXS/SANS) can provide information on the size and shape of macromolecules and nanoparticles in solution^29^. Previous SAXS and SANS studies have shown that at physiologically relevant temperature lipoproteins adopt a spherical shape with an inner core consisting of free cholesterol, cholesteryl esters and triglycerides in a disordered state^30–32^. This fatty core is then surrounded by a monolayer of phospholipid fatty acyl tails and a final outer hydrophilic layer composed of the phospholipid head groups and specific apolipoprotein (ApoB-100 for LDL and ApoA1 for HDL), as shown in Fig. 1. A specific advantage of SANS over SAXS is the large difference between the coherent scattering lengths of hydrogen and deuterium. This can be exploited to highlight specific parts of a molecule or a complex. Proteins and lipids, which are the main constituents of lipoproteins, have a natural difference in scattering length density (SLD). By changing the level of D_2_O to H_2_O in the buffer, it is possible to match the solvent SLD to that of the component of interest using contrast variation^33,34^. This means that in buffer conditions containing 5% D_2_O, the fat moieties are invisible and only the protein part of the lipoprotein will be imaged. In buffer conditions of 42% D_2_O, only the lipid component are visible, while at 100% D_2_O both components contribute to SANS data. A further way of exploiting the scattering properties of hydrogen and deuterium is through specific deuteration. Deuteration can be used to alter the SLD of a molecular moiety, or of the entire molecule, so that it can be matched out in specific buffer conditions. When studying multi-component systems such as lipoproteins interacting with other complexes (*eg* membranes and/or different membrane constituents), selective deuteration can be used in conjunction with SANS to either enhance or render invisible the respective low resolution coherent neutron scattering signals from specific components within a complex system^34,35^.

In the present study, these approaches have been used to investigate structural and compositional changes in native LDL and HDL particles upon interaction with simplified endothelial cell membranes. The cell membranes were modelled using small unilamellar liposomes prepared from a natural mixture of monounsaturated and cyclic phosphatidylcholines (dPC)^36,37^. The lipid species were selectively deuterated to be matched in SANS studies for buffer conditions of 100% D_2_O^36,37^. By probing the lipoproteins using time-resolved TR-SANS *in situ* and modelling the lipid exchange within the lipid monolayer of the lipoproteins, the lipid exchange rates for both HLD and LDL were estimated along with other structural parameters. The SANS data analysis suggested a multi-action mechanism for lipid exchange, which when combined with lipidomics, was partially attributed to the different acyl chain species present in the samples as well as the different sizes (and curvatures) of the lipoproteins. The approach established here provides an elegant way to study the effects that lipid unloading/exchange have on lipoprotein particle structure and composition.

## Results and Discussion

### SANS analytical model for lipoprotein lipid exchange

**Figure 2:**
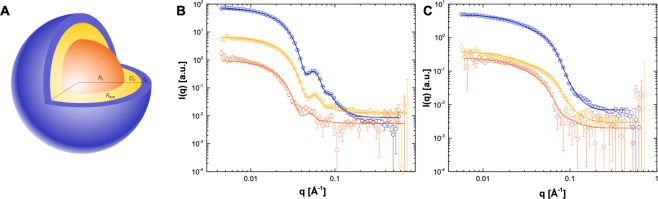
SANS model for lipoprotein particles. (**A**) Illustration of a spherical core-shell-shell particle, which is represented by an inner fat core (orange), surrounded by a monolayer of phospholipid fatty acyl tails (yellow) and a hydrophobic outer shell composed of phospholipid headgroups and apolipoproteins (blue). The total size of the particle is thus defined by the total radius at equator, *R*_*tot*_, which is a sum of the core radius *R*_*c*_, the thickness of the inner shell containing the phospholipid fatty acyl tails defined as *t*_*i*_, and the outer hydrophilic layer *t*_*o*_. (**B**) Neutron scattering intensity for LDL and (**C**) HDL particles purified from three healthy donors. Data were collected in three different contrasts; 100% D_2_O (blue open circles), 42% D_2_O (yellow open circles) and 5% D_2_O (orange open circles). Lipoprotein preparations purified from human serum were measured after size exclusion chromatography at 2 mg/ml lipid in Tris buffer at 37 °C. *I*(*Q*), SANS intensity; *Q*, momentum transfer modulus.

Previous SAXS modelling of human LDL fractions pooled from healthy donors and purified under the same conditions have shown that the core lipids in LDL are in a disordered state at 37 °C, prompting a concentric spherical core shell shape for the particle^30^. This model is illustrated in Fig. 2A, and represents the proposed structure for both LDL and HDL with an inner core consisting of cholesterol, cholesteryl esters, and triglycerides in a disordered state (orange), surrounded by a monolayer of phospholipid fatty acyl tails (yellow) and apolipoproteins (blue). Details of the modelling approach are given in the methods section.

Lipoprotein particles isolated using a previously established double purification protocol^30^ showed narrow size distributions for each lipoprotein type when analyzed by SEC (Fig. S1A). The SANS data collected at three different SANS match conditions (5%, 42% and 100% D_2_O) for both LDL and HDL are shown in Fig. 2B,C respectively, together with the best fits to the lipoprotein model schematically represented in Fig. 2A. As noted previously, at 5% D_2_O the lipid SLD is matched to the buffer and only the protein gives rise to the neutron scattering signal, at 42% only the lipids scatter neutrons as protein SLD is matched to that of the buffer whereas in 100% D_2_O the neutron scattering signal is a combination of both lipid and protein scattering. The fitting analysis showed an LDL radius of 132 Å, comparable to that reported previously for a similar preparation^30^, and an HDL radius of 62 Å, comparable with SAXS analyses by other groups^32^. The structural parameters obtained from the model fitting for both lipoprotein particles are summarized in Table 1.

**Table 1:** Parameters obtained through simultaneous model fitting to SANS data collected at three different solvent contrasts: 5% D_2_O, 42% D_2_O and 100% D_2_O. Description of the parameters are found in material and methods, and Fig. 2. *σ*_*Gaus*_, *σ*_*core*_, *σ*_*to*_ *and σ*_*ti*_ are the polydispersity values for *R*_*tot*_, *R*_*c*_, to and *t*_*i*_ respectively.

*Parameter*	*LDL*	*HDL*
*R*_*c*_ [Å]	94	28
*t*_*o*_ [Å]	22	17
*t*_*i*_ [Å]	16	17
*R*_*tot*_ [Å]	132	62
*P*	2254	309
*F*_*H*2 *O*_	0.67	0.7
*N*_*prot*_	1	2.2
*N*_*cluster*_		1.5
*σ*_*Gauss*_	0.13	0.4
*σ*_*core*_	2	5
*σ*_*to*_	5	5
*σ*_*ti*_	2	6.5

SANS data for liposomes prepared from POPC or selectively deuterated dPC prior to mixing with lipoproteins are shown in Supplementary Information (Fig. S2A,B respectively). For LDL and HDL in the absence of liposomes, the SANS data were adjusted for concentration and are shown in Fig. S2C,D, respectively. The selectively deuterated dPC liposomes showed no significant signal in the Q-range relevant for the structural analysis of lipoproteins (0.01 ≤ Q ≤ 0.35). This is especially pronounced for LDL, which demonstrated close to a 1000-fold higher SANS forward scattering than dPC-liposomes (Q = 0.01 Å^−1^) compared to the 5-fold higher signal for LDL in relation to non-deuterated POPC liposomes. For HDL, a close to a 100-fold higher SANS forward scattering intensity was observed as compared to the dPC-liposomes. Due to its small size, the SANS signal for HDL particles was lower than that of the non-deuterated POPC liposomes. Non-deuterated POPC-liposomes, on the other hand, showed both significant scattering and oscillating behavior characteristic to that of a phospholipid bilayer (Q = 0.1 Å^−1^) as well as a low degree of multi-lamellarity^38^.

The structural parameters obtained from model fitting to the SANS data collected in three D_2_O contrasts (Table 1) were further used as initial parameters for the model fitting of TR-SANS data collected after lipoproteins were mixed with liposomes composed of dPC in various concentrations. For both LDL and HDL, it was not possible to fit the exact parameters obtained from the modelling of the liposome free samples at constant concentrations (Supplementary Information Fig. S3). To obtain a similar goodness of fit, the fraction of exchange had to be released and fitted. This suggested that a certain amount of exchange had already occurred at the beginning of the measurements because of a time lag of approximately two minutes from mixing to the start of the data collection. This delay was inevitable due to the safety procedures involved with neutron experiments.

### Time-resolved SANS shows exchange in lipid monolayer of the lipoprotein

**Figure 3:**
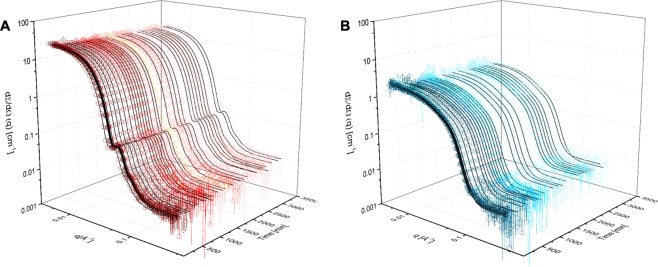
Neutron scattering intensity for lipoproteins mixed with selectively deuterated PC liposomes. (**A**) Neutron scattering intensity for LDL as a function of time after mixing with a preparation of dPC liposomes (decreasing red scale, open circles) and the corresponding fits (black lines). (**B**) Neutron scattering intensity for HDL as a function of time after mixing with a preparation of dPC liposomes (decreasing blue scale, open circles) and the corresponding fits (black lines). The data depicted are for lipoprotein:liposome ratios of 1:1 (mg/ml PC) for both LDL and HDL. All data were collected in 100% D_2_O at 37 °C.

Figure 3A shows the SANS intensity curves for a 1:1 mixture of LDL-dPC (based on the total PC concentration in mg/ml) over time (decreasing red tone) and the corresponding fits obtained through batch-fitting of the proposed model (black lines). The fitting was performed sequentially starting with the fraction of non-deuterated lipid left in particle lipid monolayer (*f*_*Hlipid*_), followed by background (Bg), polydispersity (*σ*_*gauss*_), thickness of outer layer (*t*_*o*_) and finally the number of particle clusters (*N*_*cluster*_) for the HDL datasets only. No improvements to the fits were seen when *N*_*cluster*_ was added to the LDL model - suggesting that no aggregation took place for LDL. The parameters obtained following fitting are listed in Supplementary Information (Fig. S4) together with the goodness of fit (Fig. S5). There was a clear increase in background over time for all samples studied and accounting for this in the fitting improved the modelling of both particles and all preparations. This increase in the background may reflect an increased contrast as non-deuterated lipids are incorporated randomly into the liposomes upon exchange, leading to a quasi-incoherent contribution at intermediate Q. However, as a similar trend was also observed for liposome-free preparations of lipoproteins, the increase in incoherent scattering is more likely to be due to uptake of hydrogen from the environment (Supplementary Information Fig. S2). It can also be noted that the polydispersity increased slightly with increased concentration of free dPC-liposomes while the thickness of the protein showed a slight increase during the exchange process for LDL (Supplementary Fig. S4). Releasing the thickness of the phospholipid chain region as a fitting parameter instead of the outer layer did not result in improvement of the quality of the fits. For HDL, the inclusion of an extra particle clustering parameter ***N***_***cluster***_ was necessary to improve the fit for the static data at low Q, suggesting that some degree of clustering or aggregation was observed. ***N***_***cluster***_, however, showed a slight increase for the two datasets with lower liposome concentrations only, see Fig. S4B.

**Figure 4:**
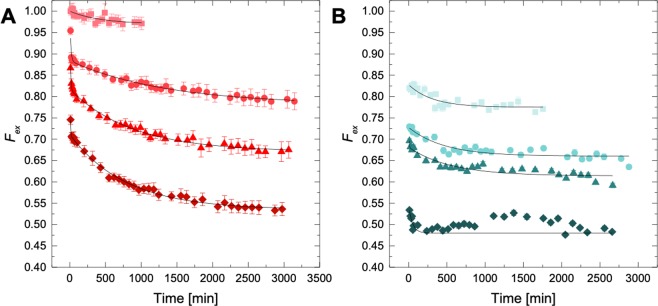
Fraction of PC exchange as a function of time. (**A**) Fraction of non-deuterated PC in an LDL lipid monolayer (*F*_*Hlipid*_) as a function of time for different lipoprotein:liposome ratios (mg/ml PC); LDL:dPC 1:0.5 mg/ml (pink), LDL:dPC 1:1 mg/ml (dark pink), LDL:dPC 1:3 mg/ml (red) and LDL:dPC 1:5 mg/ml (dark red). (**B**) Fraction of non-deuterated PC in an HDL lipid monolayer (*F*_*Hlipid*_) as a function of time for different lipoprotein:liposome ratios (mg/ml PC); HDL:dPC 1:0.5 mg/ml (cyan), LDL:dPC 1:1 mg/ml (dark cyan), LDL:dPC 1:3 mg/ml (petrol) and LDL:dPC 1:5 mg/ml (dark petrol). All data were collected in 100% D_2_O at 37 °C.

The fraction of non-deuterated lipid in the lipid monolayer decreased for both LDL and HDL (Fig. 4). For LDL, two kinetic regimes for lipid exchange could clearly be identified and were analyzed by fitting the fraction of exchange data to Eq. 7 (Fig. 4A). For HDL, on the other hand, the lipid exchange happened considerably faster than for LDL and the data could be analyzed with a single exponential function (Eq. 8) (Fig. 4B). Kinetic parameters obtained for both are listed in Table 2 and plotted in the Supplementary Information (Fig. S6).

**Table 2:** Kinetic parameters for the fraction of lipid exchange between liposomes and lipoproteins from Fig. 5.

dPC (mg/ml)	*f*_*tight*_	LDL			HDL	
		*A*	*k*_*1*_ (*min*^*−1*^)	*k*_*2*_ (*min*^*−1*^)	*f*_*tight*_	*k*_*app*_ (*min*^*−1*^)
0.5	0.97	0.03	0.00002	N.O.	0.78	0.00316
1	0.78	0.10	0.00074	0.077	0.67	0.00207
3	0.67	0.15	0.00114	0.118	0.61	0.00180
5	0.53	0.18	0.00127	0.206	0.50	0.00456

^*^N.O. Non observable.

The phospholipid concentration of the lipoprotein particle samples was held constant at 1 mg/ml in each preparation.

Supplementary Information Fig. S6 shows the fraction of tightly bound phospholipids (***f***_***tight***_) in the lipid monolayer for both LDL (red) and HDL (blue) as well as the rate constants obtained from local fitting.

For LDL, ***k***_**1**_ increased with increasing amount of PC in the liposomes reaching a saturation value at 1:3 while ***k***_***2***_ continued to steadily increase with PC concentration in the liposomes (Fig. S6B). However, the apparent rate ***k***_***app***_ did not depend significantly on the PC concentration in the liposomes for HDL (Fig. S6C). The fraction of lipids that do not undergo exchange in the observed time range, ***f***_***tight***_, probably reflects the presence of lipids with longer tail groups (see lipidomics in Fig. 6, where the main lipid species are given) as the unimer expulsion rate is critically dependent on the hydrophobic length of the amphiphiles^39–41^. For example, Nieh and coworkers^41^ have found a decrease in lipid transfer rates by more than two orders of magnitude when the acyl chain length was increased by only two carbons (from di-C14 DMPC to di-C16 (DPPC)) in bicelles and vesicles. Thus, it is likely that lipids with longer tail groups are not readily exchanged. However, ***f***_***tight***_ decreased significantly, reaching values around 0.5 upon increasing the amount of dPC for both lipoproteins. This points towards a lipid removal process that is beyond simple spontaneous exchange. It should be noted that while full exchange of PC occurs at F_H-lipid_ = 0.5 under the experimental conditions, lipid types other than PC present in the lipoproteins could also exchange and this could push the equilibrium exchange point beyond F_H-lipid_ = 0.5.

**Figure 5:**
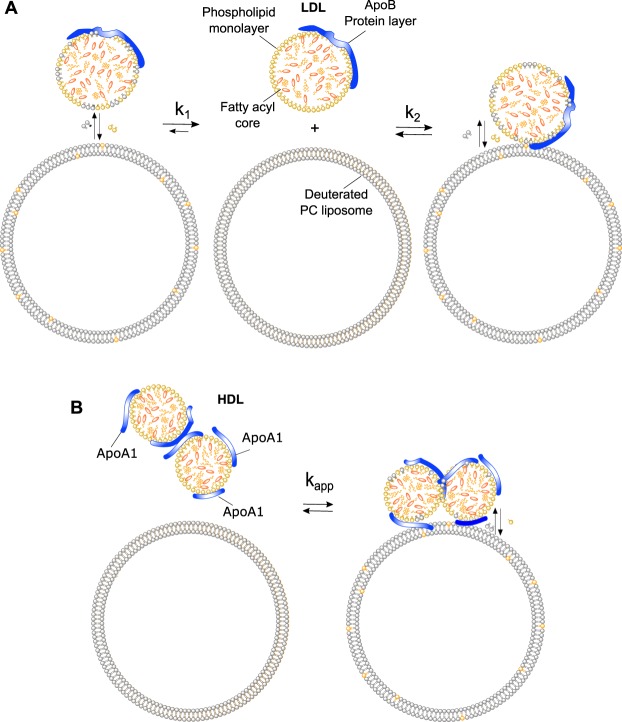
Lipid exchange between lipoproteins and deuterated PC liposomes. (**A**) Illustration of the proposed models of exchange between lipids in deuterated dPC liposomes and the phospholipid monolayer in an LDL particle. (**B**) For HDL the model of exchange includes two additional parameters: the number of apolipoprotein copies and particle clustering.

The first kinetic regime for LDL can be explained by a rate of inter-particle lipid exchange and shows the same order of magnitude as previously observed for other PC liposomes in the fluid phase^41,42^. In lipoproteins, the phospholipids are constrained to a monolayer as opposed to the bicelle, nanodisc and other vesicular structures^42^. Therefore, the second rate observed for the LDL data cannot be explained simply by lipid flip-flop, as described for liposomes^42^. Moreover, the observed second kinetic event occurs faster with increasing concentration of “free” dPC (Supplementary Information Fig. S6B). The proposed mechanism for lipid exchange between lipoproteins and cellular membranes is illustrated in Fig. 5.

**Figure 6:**
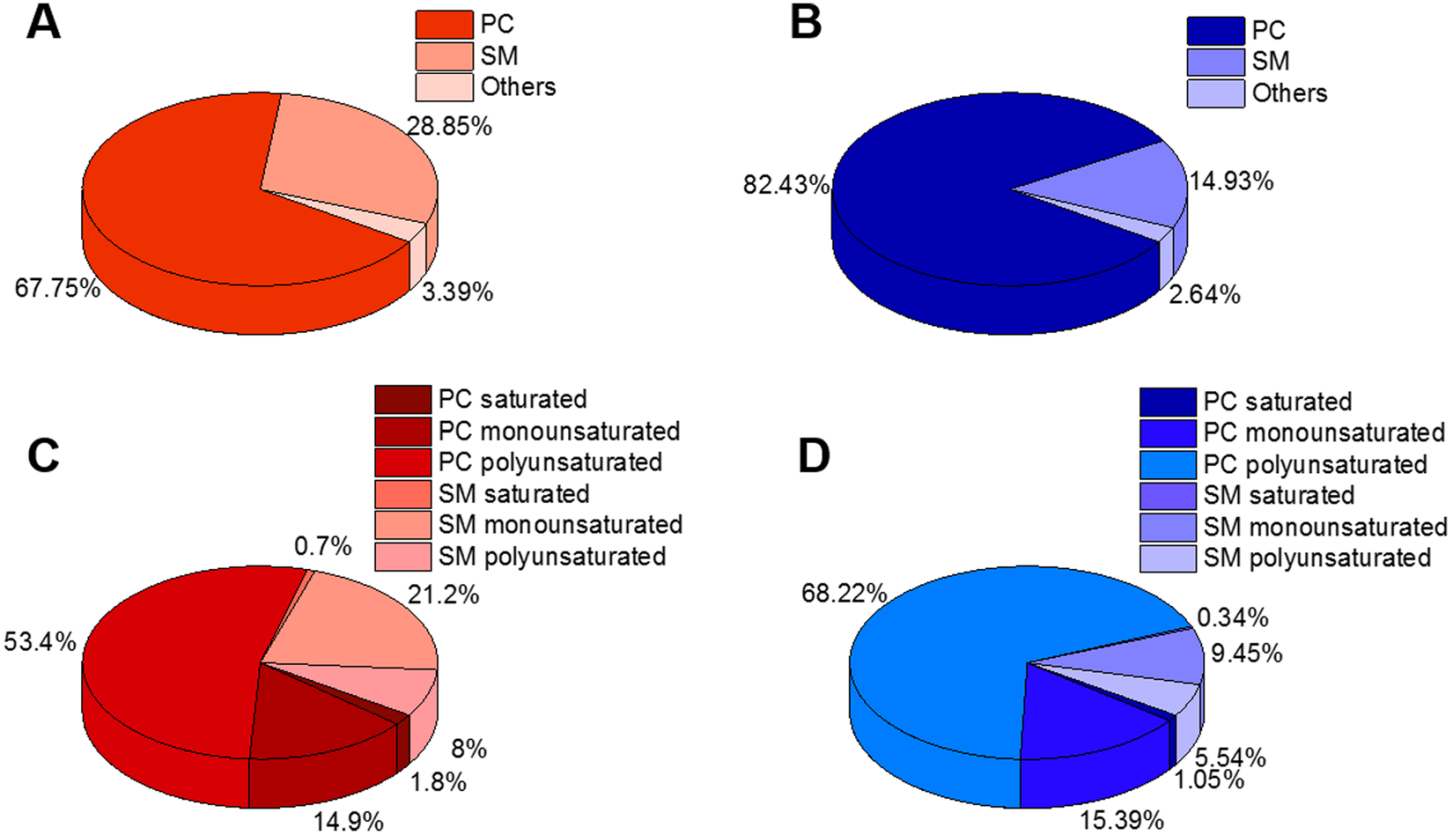
Phospholipid distribution in lipoproteins as determined by mass spectrometry. (**A**) LDL and (**B**) HDL phospholipid composition based on headgroup. (**C**) LDL and (**D**) HDL fatty acid distribution for main phospholipid species. Total lipid composition including triglycerides, cholesterol esters and free cholesterol for the two particle preparations is shown in Supplementary Information Table S1. Polyunsaturated refers to fatty acids with 2 or more double bonds.

Recently, it has been shown that both LDL and HDL adsorb to supported lipid bilayers after ~1 h incubation using quartz crystal microbalance with dissipation^28^. Hence it is proposed that lipid exchange is dependent on the mode of lipoprotein binding upon collision and further tethering to the membrane surface; the higher the concentration of liposomes, the greater the liposome surface available for lipoprotein particle adhesion. This would explain the linear increase in lipid exchange with increasing PC concentration in the liposomes. Interestingly, a small increase in the thickness of the outer layer (*t*_*o*_) was observed just at the point where the second kinetic rate starts to dominate. At the same time, there is a slight increase in polydispersity in the system (which is clearer for the mixtures containing higher liposome concentration). A change in size and polydispersity could be explained in part by a flattening of LDL upon tethering to the liposome surface to maximize contact and/or to changes in the apolipoprotein conformations that might occur upon tethering. A recent study dealing with styrene maleic acid stabilized lipid discs (SMALPs) showed a two-rate kinetic regime at phospholipid concentrations similar to those used in the present study^43^. The first kinetic regime showed a saturation with PC concentration (lipid diffusion), while the second regime showed a dependency with concentration. The latter was explained as lipid exchange due to direct contact upon particle collision without any signs of saturation. This is similar to the data for the LDL particles (Supplementary Information Fig. S6B). Here, a single kinetic regime for HDL was found that showed no dependency with respect to PC concentration (Fig. 5B). The ***f***_***tight***_ parameter however, showed that significant lipid exchange had already occurred upon the first measurement (Table 2), indicating that the initial exchange rate was too fast to be observed under these experimental conditions. Earlier, a single kinetic regime for lipid exchange was found for bicelles^41^ and apoA1 nanodiscs^44^; this was independent of concentration, and it was attributed to the inter-particle lipid exchange via aqueous solution. This is expected since the diffusion of lipids is largely controlled by the poor solubility of lipids in water and therefore exchange above this point should be constant given that the monomer concentration remains constant above this concentration limit. These experiments were however performed under conditions that inhibit bicelle fusion^45^ to ensure the single kinetic rate and saturation conditions for lipid exchange. The removal of lipids from the hydrophobic environment constitutes a high energy penalty in these systems. A higher curvature in bicelles as compared to liposomes can explain why the kinetics of lipid exchange was 2–3 orders of magnitude faster in bicelles than in liposomes^41^. Indeed, higher curvatures imply a higher hydrophobic mismatch between the different lipids species close to the edge for bicelles and by the rim of the ApoA1 protein in nanodiscs where the bilayer patch becomes less dense and well-defined^44,46^.

These highly significant differences in lipid exchange between HDL and LDL can be explained by the fact that on average each HDL particle is smaller and contains about 10 times less phospholipid than an average LDL particle (see Table 1, *P*). Hence, there are 10 times more lipoprotein particles in the HDL preparations than in the LDL preparations in the exchange experiments. This implies a larger surface area with higher curvature for HDL as compared to LDL. Experiments at lower HDL concentration were not attempted due to the low scattering intensity for this sample.

Besides diffusion limited lipid exchange, lipid transfer through collision or upon binding to specific contact sites has been proposed as the main mechanism for lipid trafficking between different organelles in cells; it has been suggested that this occurs via membrane tethering at specific contact sites^47^. Lipid exchange upon collision was shown to dominate over monomer diffusion for SMALP systems upon raising the PC concentration by a factor of 40^43^. During collision, the SMALP polymer and apolipoproteins can change conformation leading to enhanced lipid transfer. Hence the results described here suggest that lipid exchange between lipoproteins and liposomes follow a similar trend to that observed between SMALPs and apolipoprotein or Membrane Scaffold Protein (MSP)-based discs at comparable concentrations and temperature. In these conditions the lipids are expected to be in the liquid disordered phase for which exchange occurs both upon monomer diffusion, or collision and eventually tethering to the vesicle surface. It has not previously been possible to explain lipid transport processes simply by either spontaneous diffusion, vesicular trafficking or through the action of specific lipid transfer proteins^47^. While these processes could play a role to an extent, specific protein and lipid complexes have been suggested to facilitate the binding and fusion of membranes and aiding lipid exchange and transport^47^.

To understand the mode of exchange and its correlation with lipoprotein composition, both LDL and HDL were characterized for lipid composition using mass spectrometry. Figure 6 shows the lipid distribution for LDL (red) and HDL (blue) with regard to head group as well as fatty acid saturation for the main phospholipid species. The total lipid composition including triglycerides, cholesterol esters and free cholesterol for the two particle preparations is shown in Supplementary Information Table S1. Approximately two thirds of the measured phospholipid signal in this preparation of LDL were comprised of PC, followed by ~30% sphingomyelin (SM) and ~3% other phospholipids. For HDL, the PC amounted to almost 80% of total lipid, ~15% SM and almost 3% other phospholipids. In terms of fatty acyl saturation, though, the two particles showed a very similar distribution (Fig. 6C–D). The hydrophobic phospholipid monolayer thickness obtained from our lipoprotein model showed an average fatty acyl length of 17 Å. The bilayer thickness for POPC is 31 Å and DPPC is 36 Å in the fluid phase^48^ i.e. the length of the fatty acyl chains for a mixture of these two lipids would be between 15–18 Å. This fits well with the thickness of the phospholipid monolayer in our fits. It should be noted that the main lipids in these lipoprotein samples are unsaturated species. Up until now, most lipid exchange studies have used synthetic saturated lipids and have shown that differences in acyl chain length determine the exchange rates; however, the effect of unsaturation is yet to be explored^41–44,46^. The present study follows the exchange between human lipoproteins and membranes composed of natural lipids carrying long, unsaturated fatty acids. The data obtained shows rates that are slightly slower than those observed for systems composed of the shorter, synthetic, unsaturated dimyristoylphosphatidylcholine (DMPC) measured at lower temperatures (30 °C)^43^. This is to be expected given that the transport of a longer and bulkier lipid carries a higher energy penalty. Moreover, the presence of other lipids, such as SM, is expected to affect the apparent rate of exchange since *in vivo* studies suggest that SM in lipoproteins exchanges more slowly than PC^14^. Finally, it is expected that the lipid exchange that occurs due to lipoprotein collision and tethering to endothelial cell membranes is faster since there is a laminar blood flow in the arteries – despite the disruption at arterial branch points and curvatures where atherosclerotic plaques and lesions tend to locate^49^. The methods described here open up the opportunity to follow lipid exchange in the presence of either laminar or turbulent flow by – for example – introducing shear forces during data collection.

## Conclusions

Based on the use of SANS and selective deuteration, a reliable and robust method is described that allows the effects of lipid exchange on lipoprotein structure to be studied. The data suggests that, besides monomer diffusion controlled exchange, lipid exchange happens through collision and tethering, and that the apolipoprotein type plays a role in the exchange indirectly through the mode of binding to the cell-membrane surfaces. Moreover, this method allows the composition of the liposome lipid donor to be controlled and therefore permits systematic studies of specific lipid effects on exchange and transfer events. Such studies are now possible not only between lipoproteins but also between other organelles in the cell and other complex biological protein-lipid systems. The development of faster neutron instrumentation, in combination with tailor-deuteration of natural mammalian phospholipids^36,50–53^ and sterols^54^, offers the prospect of tackling even more complex phenomena^48–51^. For lipoprotein dynamics and their role in atherosclerosis, this approach can lead to novel insights to the roles of different lipid species (having different acyl chain compositions), as well as the degree to which lipid oxidation affects lipid uptake and exchange. Finally, the lipoprotein preparations described in this study are quite polydisperse in composition (Fig. 6) despite being monodisperse in size (Fig. 2 and Table 1). By performing detailed studies with sub-fractions isolated on the basis of their apolipoprotein composition (lipoproteins that are enriched with specific apolipoproteins) as well as through comparison with simplified artificial reconstituted lipoproteins, it is possible now to consider unravelling the role of specific apolipoproteins in complex systems. Such studies will be of considerable value for an understanding of the molecular mechanisms underlying plaque buildup – in particular the structures of the lipoprotein particle and the artery wall during the interactions that are thought to lead to the onset of atherosclerosis.

## Materials and Methods

### Materials

D_2_O (99.9% deuterated, Sigma Aldrich) was provided by ISIS Neutron and Muon Source. Ultrapure Milli-Q (MQ) water was used for all cleaning procedures and solvents. Tris buffer was prepared by dissolving a tablet of 50 mM Tris, 150 mM NaCl, pH 7.4 as specified by the producer (Sigma-Aldrich) in either D_2_O or H_2_O. Lipid standards and 1-palmitoyl-2-oleoyl-*sn*-glycero-3-phosphocholine (POPC) were purchased from Avanti Polar Lipids (Alabaster, AL).

### Purification of lipoproteins from human plasma

Native human LDL and HDL were purified from plasma from three healthy donors as previously described^30^. After an initial purification using ultra-centrifugation, fractions were collected at 1.019–1.063 g/ml for LDL and 1.063–1.210 g/ml for HDL and further purified using Size Exclusion Chromatography (SEC) on a Superose 6 Increase 10/300 GL column (GE Healthcare) after a buffer exchange to Tris buffer (Sephadex G25 PD-10 desalting column) from the storage buffer (50% w/v sucrose, 150 mM NaCl, 24 mM EDTA, pH 7.4). The SEC was performed at room temperature and the samples stored at 4 °C and used within one week. The chromatograms for both HDL and LDL are shown in Supplementary Information (Fig. S1). The concentration of the total protein component of each lipoprotein was determined by the Bradford method using bovine serum albumin as standard^55^. Phosphatidylcholine was determined using the phosphatidylcholine assay kit according to the manufacturer’s instructions (Sigma Aldrich) prior to all neutron measurements. The total cholesterol and triglyceride content were determined by colorimetric assays (Pointe Scientific) as previously described^30^. The composition of the lipoprotein fractions studied is given in Table S1. The precise same samples of LDL and HDL fractions, n = 1 each pooled from three doners (see above) that were utilised for the SANS study, were then subjected to the mass spectrometry based lipidomics.

### Biosynthesis of deuterated phosphatidylcholine

Selectively deuterated mixed acyl phosphatidylcholine (dPC) was produced in a modified *E*. *coli* strain grown in 100% deuterated minimal medium supplemented with deuterated glycerol (C_3_D_8_O_3_) and partially deuterated choline chloride (trimethyl-d9, 98%; Eurisotop) as previously described^50^. Total lipids were extracted using a modified method of Bligh and Dyer^56^ and purified based on headgroup polarity using silica-gel column chromatography with varying ratios of chloroform and methanol as previously described^50^. The lipids were dried and stored as lipid films under N_2_ and at −20 °C. Phospholipid composition was characterized by thin-layer chromatography (TLC) through comparison with known standards as previously described^36,50^, the lipid amounts estimated through measurement of total phosphorus according to Rouser *et al*.^57^. Purified PC was assayed for fatty acid composition using matrix-assisted laser desorption/ionization time-of-flight (MALDI–TOF) mass spectrometry (MS) as described before^50^.

### Preparation of liposomes

Liposomes were formed by resuspending lipid films in Tris buffer prepared in 100% D_2_O at 10 mg/mL for 30 min. The suspension was vortexed a couple of times followed by 11 times extrusion through 100 nm polycarbonate filters (Avanti Polar Lipids). The PC concentration of the final extruded suspension was determined using a phosphatidylcholine assay kit according to the manufacturer’s instructions (Sigma Aldrich) immediately prior to the neutron measurements.

### Design of the kinetics lipid mixing study

The kinetics study was designed based on total PC concentration in the lipoproteins (estimated to 6 mg/ml after SEC purification) and in the deuterated liposomes. The concentration ofthe lipoproteins was kept constant while the concentration of liposomes was varied to a ratio of 1:0.5, 1:1, 1:3 and 1:5 liposome to lipoprotein (PC mg/ml). Prior to the mixing experiments, SANS data for LDL, HDL were collected in three different solvents (100% D_2_O, 42% D_2_O and 5% D_2_O), and fresh preparations of deuterated and non-deuterated liposomes (made from POPC) were measured in 100% D_2_O. For kinetic measurements, the lipoprotein stocks were mixed with an equal volumes of particular liposome suspension. The kinetic experiments were performed at 37 °C in 100% D_2_O buffer, where the liposomes showed no significant scattering over the chosen q-range studied (see Supplementary Information Fig. S2).

### SANS data collection

All reported SANS data were collected on the SANS2D small-angle diffractometers in the time-of-flight mode, at the ISIS Pulsed Neutron Source (STFC Rutherford Appleton Laboratory, Didcot, U.K.)^58,59^. Data were collected at 37 °C in flat rectangular Hellma quartz cuvettes with a path length of 1 or 2 mm, depending on the D_2_O content, in order to optimize signal-to-noise and minimize incoherent background and multiple-scattering effects. A simultaneous Q-range of 0.0045–0.7 Å^−1^ was achieved utilizing an incident wavelength range of 1.75–16.5 Å and employing an instrument set up of L1 = L2 = 4 m, with the 1 m^2^ detector offset vertically 60 mm and sideways 100 mm. Q is defined as:

1 $$Q=\frac{4\pi \,\sin \,\frac{\theta }{2}}{\lambda }$$

where θ is the scattered angle and λ is the incident neutron wavelength. The beam diameter was 8 mm. Each raw scattering data set was corrected for the detector efficiencies, sample transmission and background scattering and converted to scattering cross-section data (∂Σ/∂Ω vs. Q) using the instrument-specific software^60^. These data were placed on an absolute scale (cm^−1^) using the scattering from a standard sample (a solid blend of hydrogenous and perdeuterated polystyrene) in accordance with established procedures^61^.

### Derivation of the SANS analytical model for lipoprotein lipid exchange

The total size of the particle is thus defined by the total radius at equator: ${R}_{tot}={R}_{c}+{t}_{i}+{t}_{o}$, where *R*_*c*_ is the core radius, while *t*_*i*_ and *t*_*o*_ are the thickness of the inner shell containing the phospholipid fatty acyl tails and the outer hydrophilic layer *t*_*o*_, respectively.

The total scattering intensity *I*(*q*) can thus be written as:

2 $$\begin{array}{rcl}I(q) & = & \frac{\phi }{{V}_{dry}}S(q)\cdot (A{(q)}_{core}\cdot {\rm{\Delta }}{\rho }_{core}\cdot {V}_{core}\\  &  & +\,A{(q)}_{innershell}\cdot {\rm{\Delta }}{\rho }_{innershell}\cdot {V}_{innershell}\\  &  & +\,A{(q)}_{outershell}\cdot {\rm{\Delta }}{\rho }_{outershell}\cdot {V}_{outershell}\,{)}^{2}\end{array}$$

Where S(q) is the structure factor which was negligible for LDL and thus S(q) ≈ 1, *φ* is the volume fraction. The total “dry” volume of a single lipoprotein particle is defined as:

$${V}_{dry}={V}_{core}+{V}_{innershell}+{f}_{dry}\cdot {V}_{outershell}^{d}$$

With the volume of the inner hydrocarbon core, *V*_*core*_, defined as: ${V}_{core}=\frac{4}{3}\cdot \pi \cdot {R}_{c}^{3}$, the volume of the inner phospholipid fatty acyl tail shell, *V*_*innershell*_, is defined as: ${V}_{innershell}=\,\frac{4}{3}\cdot {\rm{\pi }}\cdot ({({R}_{c}+{t}_{i})}^{3}-{R}_{c}^{3})$ and the volume of the outer hydrophilic shell, *V*_*outershell*_, containing the phospholipid head groups and protein, is defined as:

$${V}_{outershell}=\frac{4}{3}\pi \cdot ({R}_{tot}^{3}-{({R}_{c}^{}+{t}_{i})}^{3})$$

In order to accommodate for the exchange of phospholipid in the outer shell, the fraction of material in the outer shell is described by:

$${f}_{dry}=\frac{({N}_{protein}\cdot {V}_{protein}+P\cdot {V}_{lipidhead})}{{V}_{outershell}}$$

where *N*_*protein*_ is the number of proteins, *V*_*protein*_ is the volume of the specific apolipoprotein, *P* is the number of phospholipids and *V*_*lipidhead*_ is the volume of the lipid headgroups.

The contrasts for the different layers are defined by

$${\rm{\Delta }}{\rho }_{core}={\rho }_{core}-{\rho }_{solvent}$$

$${\rm{\Delta }}{\rho }_{innershell}={\rho }_{Htail}\cdot {f}_{Hlipid}+{\rho }_{Dtail}\cdot (1-{f}_{Hlipid})-{\rho }_{solvent}$$

where ${f}_{Hlipid}$ is the fraction of hydrogenated lipids in the phospholipid monolayer. Further,

3 $$\begin{array}{rcl}{\rm{\Delta }}{\rho }_{outtershell} & = & (\frac{{N}_{protein}\cdot {V}_{protein}}{{N}_{lipid}\cdot {V}_{lipidhead}+{N}_{protein}\cdot {V}_{protein}}\cdot {\rho }_{protein}\\  &  & +\,\frac{P\cdot {V}_{lipidhead}}{{N}_{lipid}\cdot {V}_{lipidhead}+{N}_{protein}\cdot {V}_{protein}}\cdot {\rho }_{lipidhead})\cdot {f}_{dry}\\  &  & +\,{\rho }_{solvent}\cdot (1-{f}_{dry})-{\rho }_{solvent}\end{array}$$

Where *ρ*_*core*_ and *ρ*_*protein*_ are the scattering length density of the particle core and the protein respectively.

The scattering amplitude of the concentric layers is described by:

$$A{(q)}_{i}=\{\begin{array}{c}a(q,{R}_{c})\cdot \exp (-\,\frac{{q}^{2}{{\sigma }_{c}}^{2}}{2}),\,{\rm{for}}\,{\rm{inner}}\,{\rm{core}}\\ ({({R}_{c}+{t}_{i})}^{3}a(q,{R}_{c}+{t}_{i})\cdot \exp (-\,\frac{{q}^{2}{{\sigma }_{i}}^{2}}{2})-{R}_{c}^{3}a(q,{R}_{c})\cdot \exp (-\,\frac{{q}^{2}{c}^{2}}{2})/({({R}_{c}+{t}_{i})}^{3}-{R}_{c}^{3}),{\rm{for}}\,{\rm{lipid}}\,{\rm{layer}}\\ ({({R}_{c}+{t}_{i}+{t}_{o})}^{3}a(q,{R}_{c}+{t}_{i}+{t}_{o})\cdot \exp (-\,\frac{{q}^{2}{{\sigma }_{o}}^{2}}{2})-{({R}_{c}+{t}_{i})}^{3}a(q,{R}_{c}+{t}_{i})\cdot \exp (-\,\frac{{q}^{2}{{\sigma }_{i}}^{2}}{2})/({({R}_{c}+{t}_{i}+{t}_{o})}^{3}-{({R}_{c}+{t}_{i})}^{3})),\\ {\rm{for}}\,{\rm{j}}={\rm{outer}}\,{\rm{core}}\end{array}$$

4 $${\rm{Where}}\,{a}(q,x)=3\cdot (\sin (R\cdot x)-R\cdot x\cdot \,\cos (R\cdot x))/{(q\cdot x)}^{3}$$

For HDL particles, which contain several different proteins and vary in the number of protein copies, it was necessary to take into account the observed attractive interactions by including a structure factor corresponding to ${N}_{cluster}$ randomly connected lipoproteins with bond length D which was added to Eq. 2:

5 $${S}_{{N}_{cluster}}(x)=\frac{2}{1-(\frac{sinx}{x})}-1-\frac{2[1-{(1-(\frac{sinx}{x}))}^{{N}_{cluster}}]}{{N}_{cluster}(1-{(\frac{sinx}{x})}^{2})}(\frac{sinx}{x})$$

Where *x* = D·q, ${N}_{cluster}$ should have only integer values, and therefore for any noninteger values ${N}_{cluster}\,\,$was weighted by taking a linear combination of $|{N}_{cluster}|$ and $|{N}_{cluster}|+1$. With $p={N}_{cluster}-|{N}_{cluster}|$, ${S}_{{N}_{cluster}}(x)$ can then be expressed by^62^:

6 $${S}_{{N}_{cluster}}(x)=(1-p)\cdot {S}_{|{N}_{cluster}|}(x)+p\cdot {S}_{|{N}_{cluster}|+1}(x)$$

### Model fitting of lipid exchange

Scattering length densities of the different lipoprotein components used for the model fitting are summarized in Supplementary Table S2. The models described above were implemented into QtiKWS^63^ and the theoretical scattering signals for LDL and HDL were calculated and fitted to the experimental data collected at different concentrations of D_2_O simultaneously. The parameters, including lipoprotein size and shape, obtained through contrast variation (Table 1) were then used as initial parameters for the subsequent model-fitting of the particles to describe the time-dependent scattering curves during the lipid exchange events. The data was fitted successively and using the batch-fit mode available through QtiKWS. First, the fraction of non-deuterated lipid left in the lipoprotein monolayer, *F*_*Hlipid*_, was used as the only free parameter but this did not give a satisfactory fit. Then, and the number of free fitting parameters was increased until a satisfactory fit was obtained. These parameters included background (*Bg*), polydispersity (***σ***_***gauss***_), thickness of outer layer (***t***_***o***_) and for the HDL also number of particle clusters (***N***_***cluster***_) in a sequential and systematic manner until a satisfying goodness of fit was achieved. Fitting the thickness of the lipid chain region resulted in no improvement of the fit quality.

The kinetic rates for LDL were fitted using a double exponential function:

7 $${f}_{Hlipid}(t)={f}_{tight}+A\cdot {e}^{(-\frac{t}{\tau 1})}+(1-A-{f}_{tight})\cdot {e}^{(-\frac{t}{\tau 2})}$$

where $\,{f}_{tight}$ is the fraction of lipids that exchange very slowly, ${k}_{1}=\frac{1}{{\tau }_{1}}$ and ${k}_{2}=\frac{1}{{\tau }_{2}}$ and *A* indicates the probability for the process with *k*_1_ rate to occur. The “pseudo” first order rate constant ${k}_{app}$ for HDL was determined by fitting a single exponential function to the obtained data using:

8 $${f}_{Hlipid}(t)=\,{f}_{tight}+(1-{f}_{tight})\cdot {e}^{(-\frac{t}{\tau })}$$

where $${k}_{app}=\frac{1}{{\tau }_{}}$$.

### Lipidomic analysis

Lipids from purified lipoprotein particles were extracted using the single-phase approach^64,65^. Briefly, 10 µl of either of the lipoprotein solution were thoroughly mixed with 100 µl butanol/methanol (1:1) containing internal standards and ultra-sonicated for 10 min. Protein fractions were precipitated by centrifugation for 20 min at 4 °C at 20,000 × g and the supernatant was transferred into 250 µl deep well micro titer plates, the extraction repeated once without the added standards, and the supernatants combined. The lipids were dried under vacuum then subsequently dissolved in 100 µl butanol/methanol 1:1. The targeted LC-MS/MS analysis was carried out on an electrospray triple quadrupole mass spectrometer coupled with an ultra-high-pressure liquid chromatography system (MS AGILENT QQQ 6460, UHPLC AGILENT 1290). The separation was carried out on an AGILENT C18 column (ZORBAX, 1.7 µm particle size, 100 × 2.1 mm) at a flow rate of 400 µl/min with a gradient from 60% A (60% Milli-Q Water, 40% acetonitrile with 10 mM ammonium formate) to 100% B (90% isopropanol, 10% acetonitrile with 10 mM ammonium formate) in 5 mins. Lipids were analyzed in the targeted Multiple Reaction Monitoring (MRM) approach covering the lipids listed in the results. The obtained raw data were processed using MassHunter Quantiative Analysis Ver 7.0 (AGILENT) and the obtained integration data verified manually.

### Illustrations

All illustrations were made using the open source vector graphics software InkScape Version 0.91^66^.

### Ethics statement

Studies investigating human HDL and LDL involve lipid extraction from plasma obtained from voluntary healthy blood human donors that have provided a written consent. Plasma is obtained from Clinical immunology and transfusion medicine, Skåne University Hospital, Sweden and conducted in accordance with Swedish law. The plasma is anonymised and outdated and can therefore not be used for transfusion of patients.

## Supplementary information

Supplementary data

## Data Availability

All data are available in the main text or the Supplementary Materials.
