# Supplementary material for: Time-resolved small-angle neutron scattering as a probe for the dynamics of lipid exchange between human lipoproteins and naturally derived membranes
Source: Sci Rep. 2019 May 20;9:7591. doi: 10.1038/s41598-019-43713-6 (PMC6527577; doi:10.1038/s41598-019-43713-6)
Supplement: Supplementary file 1 — Supplementary data [file 41598_2019_43713_MOESM1_ESM.pdf]

Supplementary Information

Time-resolved small-angle neutron scattering as a probe for the dynamics of lipid exchange between human lipoproteins and naturally derived membranes.

Selma Maric,[1*] Tania Kjellerup Lind,[1] Manfred Roman Raida,[2] Eva Bengtsson,[3] Gunilla Nordin Fredrikson,[3†] Sarah Rogers,[4] Martine Moulin,[5] Michael Haertlein,[5] V. Trevor Forsyth,[5] Markus Wenk,[2] Thomas Günther Pomorski,[6,7] Thomas Arnebrant,[1] Reidar Lund[8] and Marité Cárdenas.[1*]

Affiliations:

[1]Dept. of Biomedical Science, Malmö University, Per Albin Hanssons väg 35, 205 02 Malmö, Sweden.

[2]Singapore Lipidomics Incubator (SLING), Life Sciences Institute, National University of Singapore.

[3]Dept. of Clinical Sciences, Lund University, Jan Waldenströms gata 35, CRC, Box 50332, 212 13 Malmö, Sweden.

[4]ISIS Science and Technology Facilities Council, Harwell Science and Innovation Campus, Chilton, Didcot, Oxfordshire OX11 0QX, United Kingdom

[5]Life Science Group, Institut Laue Langevin, 6, rue Jules Horowitz, BP 156, F-38042 Grenoble Cedex 9, France.

[6]Dept. of Plant and Environmental Sciences, University of Copenhagen, Thorvaldsensvej 40, 1871 Frederiksberg C, Denmark.

[7]Dept. of Molecular Biochemistry, Ruhr University Bochum, Faculty of Chemistry and Biochemistry, 44780 Bochum, Germany

[8]Dept. of Chemistry, University of Oslo, Blindern, 0315 Oslo, Norway

*Correspondence to: marite.cardenas@mau.se and selma.maric@mau.se
[†]Deceased on the 20th of January 2019

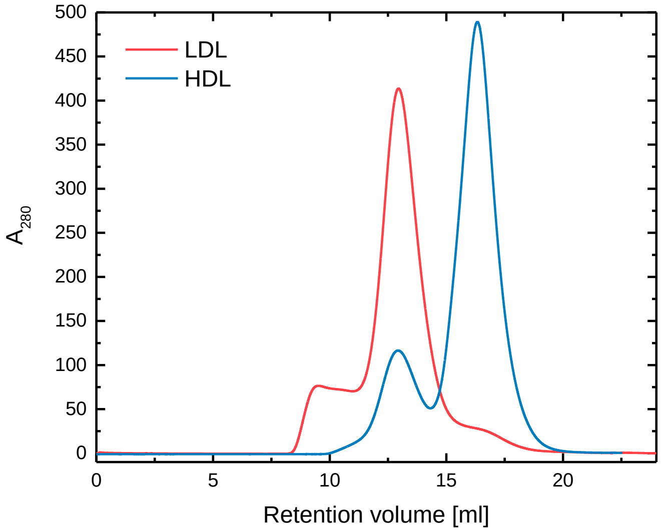

Supplementary Figure S1. Lipoprotein purification and characterization. Size Exclusion Chromatography for LDL (red) and HDL (blue**)**. The samples were purified from fresh plasma pooled from three healthy men in Tris buffer, using a Superose 6 column (GE Healthcare). The protein concentration was determined to 0.54 and 1.04 mg/ml for the pooled fractions corresponding to LDL and HDL, respectively. Details on the purification are shown in methods section based on a previously established double purification protocol.[1]

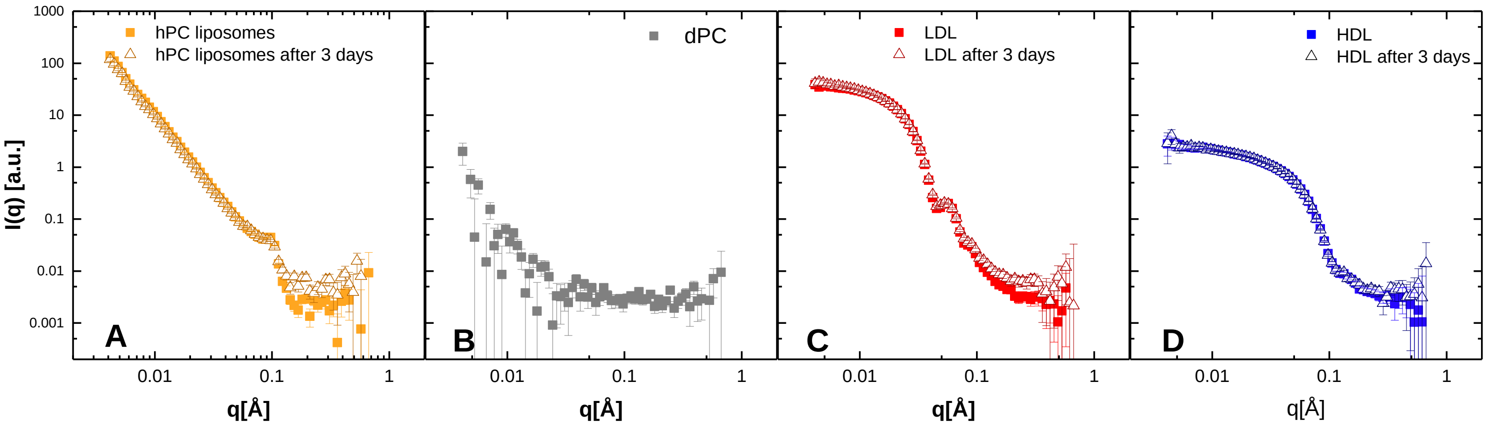

Supplementary Figure S2. Characterization of the individual lipoproteins and liposomes. SANS intensity profiles for: **(A)** Non-deuterated POPC liposomes at start (yellow squares) and end of the experiments (open yellow triangles). **(B)** Deuterated dPC liposomes (grey squares). **(C)** LDL particles at start of measurements (closed red squares) and after three days (open red triangles). $I(q)$, SANS intensity; q, momentum transfer modulus. **(D)** HDL particles at start of measurements (closed blue squares) and after three days (open blue triangles). $I(q)$, SANS intensity; q, momentum transfer modulus.

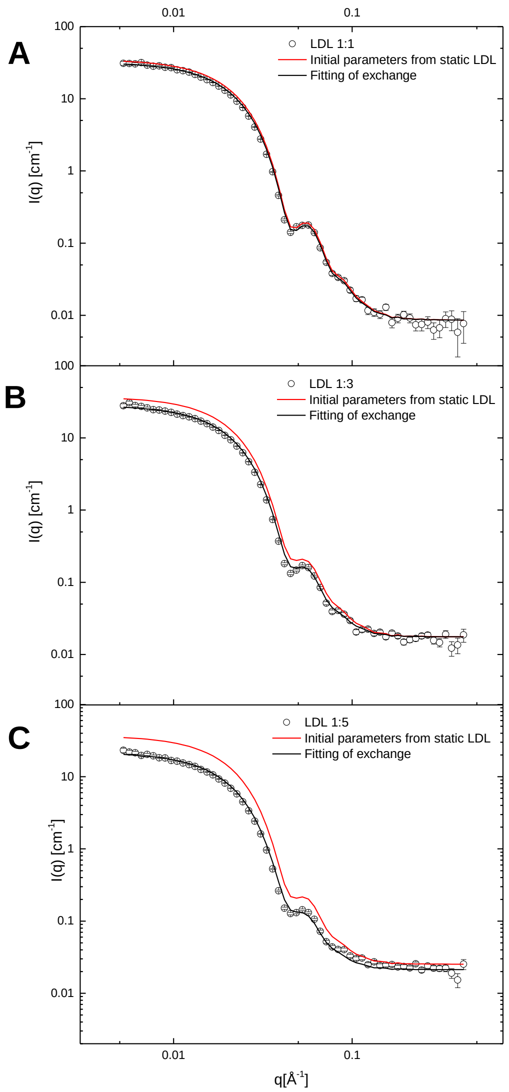

Supplementary Figure S3. Model fitting of LDL after mixing with liposomes. SANS intensity profiles for: **(A)** LDL together with dPC liposomes (open circles) at a ratio of 1:1 (PC mg/ml), **(B)** LDL together with dPC liposomes (open circles) at a ratio of 1:3 (PC mg/ml), **(C)** LDL together with dPC liposomes (open circles) at a ratio of 1:5 (PC mg/ml). Model fits at zero level of lipid exchange are shown as red lines and fraction of exchange fitted as black lines.

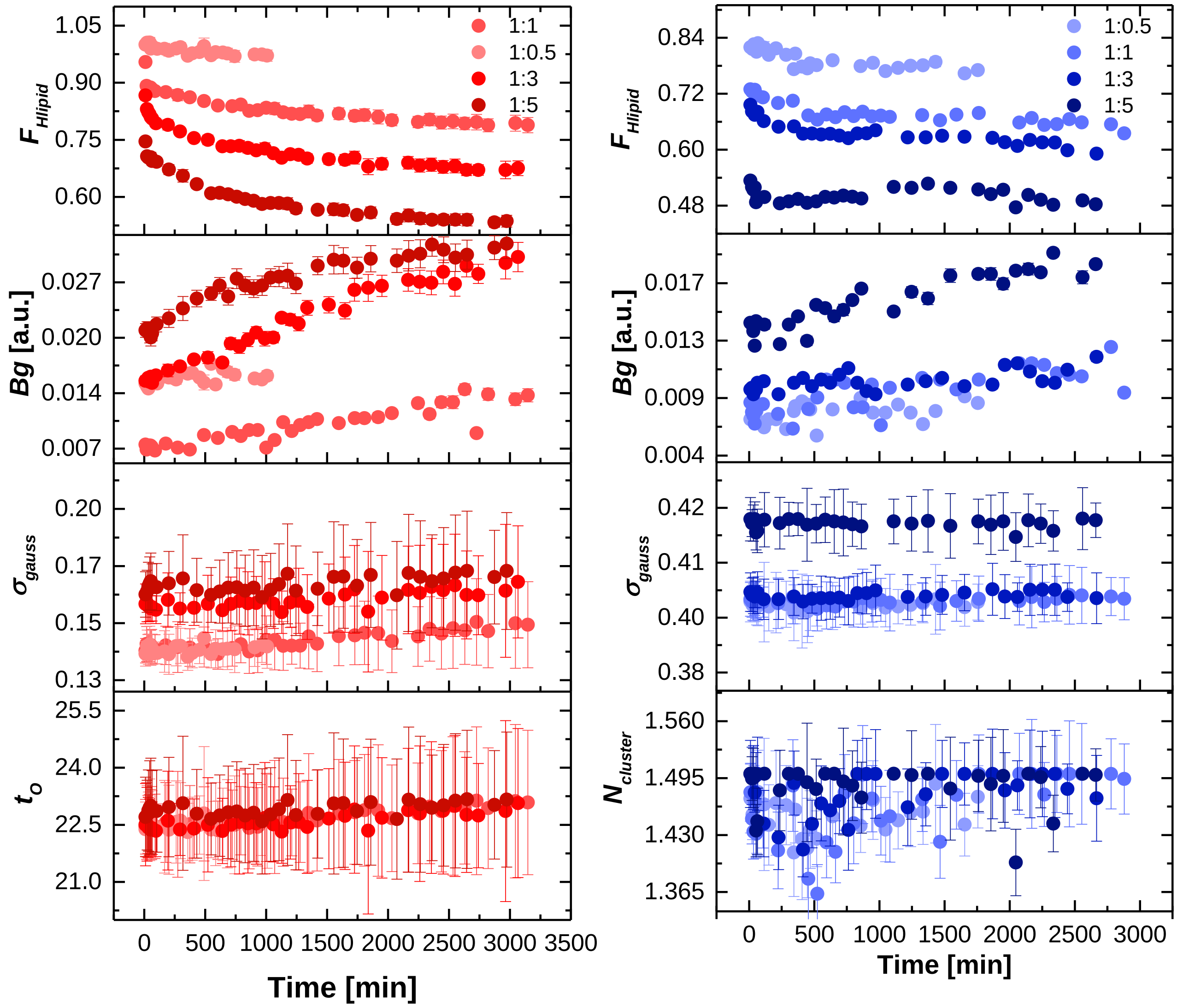

Supplementary Figure S4. Fitting parameters for LDL (A) and HDL (B) during lipid exchange with natural PC extracts. From top to bottom: Fraction of non-deuterated PC lipid left in particle lipid monolayer (***F_{Hlipid}*)**, background (***Bg***), polydispersity (***σ_{gauss}***), Thickness of the protein layer (***$T_{protein}$***) and number of particle clusters (***$N_{cluster}$***). The different lipoprotein:liposome ratios (in mg/ml PC) are as follows: LDL:dPC 1:0.5 mg/ml (pink), LDL:dPC 1:1 mg/ml (dark pink), LDL:dPC 1:3 mg/ml (red) and LDL:dPC 1:5 mg/ml (dark red). And for HDL: HDL:dPC 1:0.5 mg/ml (cyan), LDL:dPC 1:1 mg/ml (dark cyan), LDL:dPC 1:3 mg/ml (blue) and LDL:dPC 1:5 mg/ml (dark blue).

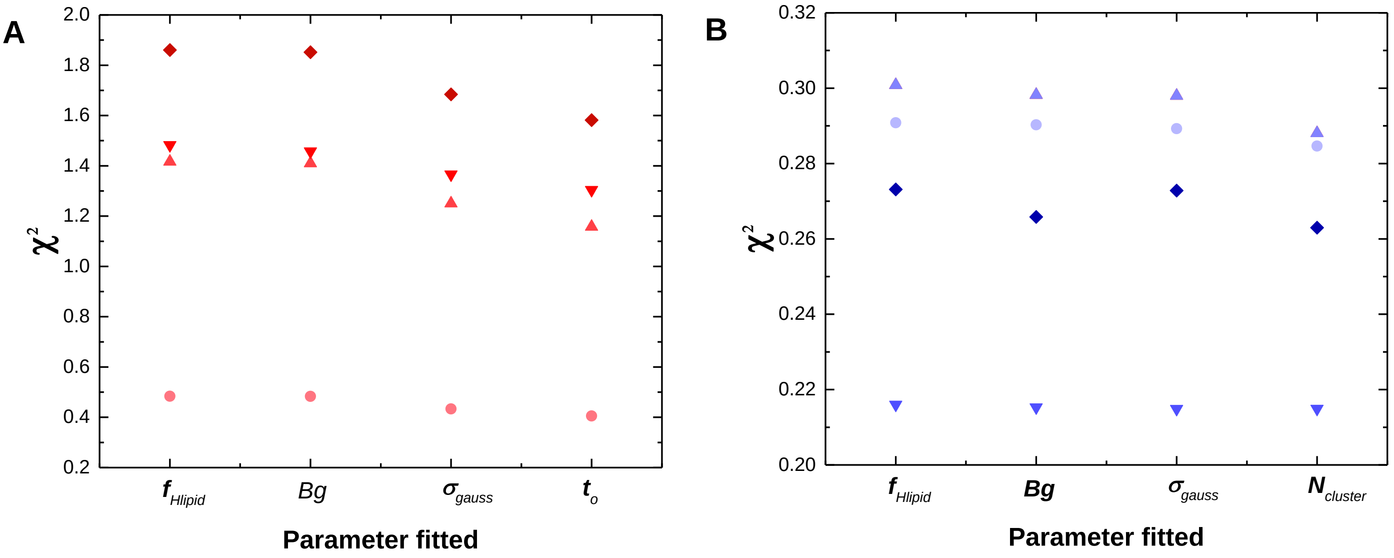

Supplementary Figure S5. Goodness of fit (χ^2) for the two models. (A) Goodness of fits for the concentric spherical three shell LDL model and lipid exchange between PC liposomes and the LDL phospholipid monolayer only for. **(B)** Goodness of fits for the concentric spherical three-shell model for HDL that includes particle clustering and variable number of apolipoprotein copies. The data was fitted in a sequential manner starting with fraction of exchange of non-deuterated lipid (f_{Hlipid}), followed by background (***Bg***), polydispersity (σ_{gauss}), thickness of protein layer ($T_{protein}$) and for HDL also number of particle clusters ($N_{cluster}$) for different lipoprotein:liposome ratios (in mg/ml PC) are as follows: LDL:dPC 1:0.5 mg/ml (pink), LDL:dPC 1:1 mg/ml (dark pink), LDL:dPC 1:3 mg/ml (red) and LDL:dPC 1:5 mg/ml (dark red). And for HDL: HDL:dPC 1:0.5 mg/ml (cyan), LDL:dPC 1:1 mg/ml (dark cyan), LDL:dPC 1:3 mg/ml (blue) and LDL:dPC 1:5 mg/ml (dark blue).

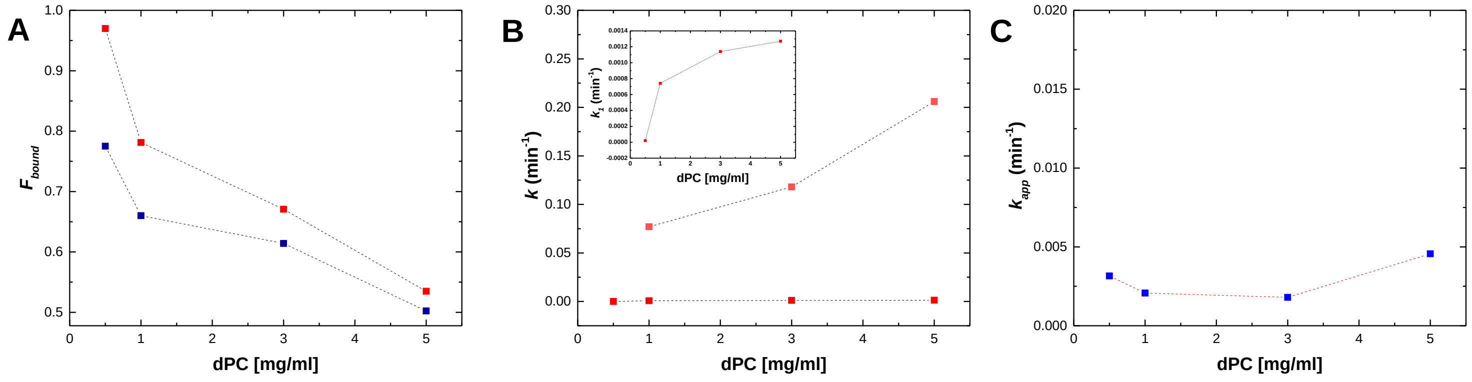

Supplementary Figure S6. Lipid exchange parameters derived from local fitting of the TR-SANS obtained parameters (Fig.4 Main Text). (A) Fraction of bound (unexchanged) lipids for LDL (red) and HDL (blue). **(B)** LDL lipid exchange constants k_1 (red) and k_2 (light red) obtained from local fits of SANS obtained parameters to Equation 7. *Insert*: Zoomed view of k_1. **(C)** Apparent rate constant k_{app} for HDL obtained from local fit of SANS parameters to Eq. 8.

Supplementary Table S1: Lipoprotein composition after Size Exclusion Chromatography, as determined by colorimetric assays (see method section).

Lipoprotein component	LDL [%w/w]	HDL [%w/w]
Protein	25.3 ± 0.6	62.9 ± 3.2
Phospholipid	18.0 ± 0.5	15.9 ± 0.8
Total cholesterol	50.7 ±1.3	17.4 ± 0.9
Triglycerides	6.0 ± 0.5	3.8 ± 0.2

Supplementary Table S2: Fitting parameters

Parameter	LDL	HDL	Liposome
	SLD [10^{-6} Å^{-2}][a]		
$\rho_{core}{}^{b}$	0.25	0.25	
$\rho_{Htail}{}^{c}$	-0.30	-0.30	
$\rho_{HheadL}{}^{c}$	1.8	1.8	
$\rho_{protein}$	3.09	3.10	
ρ_{Dtail}			6.07
ρ_{DheadL}			6.07
	Particle specific volume [Å^3]		
V_{tailL}	930	930	
V_{headL}	320	320	
$V_{protein}$	635505	37798	
D		120	
	Fitted parameters		
$\rho_{outershell}$ [10^{-6} Å^{-2}]	5.03	5.33	
R_c [Å]	94	28	
t_i [Å]	16	16.9	
t_0 [Å]	22	17	
R_{tot} [Å]	132	61.9	
N_{lipid}	2254	309	
F_{H2O}	0.67	0.70	
N_{prot}	1	2.2	
$N_{cluster}$		1.50	
	Polydispersity/error terms		
σ_{Gauss}	0.13	0.4	
σ_{core}	2	5	
σ_{to}	5	5	
σ_{ti}	2	6.5	

[a]The SLDs were calculated from the partial specific volumes and nuclear scattering lengths using the SLD calculator provided with the Motofit[2] package (IgorPro).

[b] The SLD of the fatty core was approximated to $0.25 \cdot 10^{-6}$ Å^{-2} based on the SLD calculated for an example triglyceride containing palmitic acid, oleic acid and alpha-linoleic acid ($C_{55}H_{98}O_6$,$0.25 \cdot 10^{-6}$ Å^{-2}), free cholesterol ($C_{27}H_{46}O$, $0.22 \cdot 10^{-6}$ Å^{-2}) and cholesteryl linoleate ($C_{45}H_{76}O_2$, $0.26 \cdot 10^{-6}$).

[c]Using a partial specific volume of a POPC tail and head group.[3]
[d]The partial specific volumes of the proteins were calculated using Protparam[4] and the ApoB/ApoA1 FASTA sequences available via UniProt.[5] The SLDs of the proteins were then

calculated using the Biomolecular SLD Calculator available online via the Science & Technology Facilities Council.[6]

References

1 Maric, S., Lind, T. K., Lyngso, J., Cardenas, M. & Pedersen, J. S. Modeling Small-Angle X-ray Scattering Data for Low-Density Lipoproteins: Insights into the Fatty Core Packing and Phase Transition. *ACS Nano* **11**, 1080-1090, doi:10.1021/acsnano.6b08089 (2017).

2 Nelson, A. Co-refinement of multiple-contrast neutron/X-ray reflectivity data using MOTOFIT. *J Appl Crystallogr* **39**, 273-276, doi:10.1107/S0021889806005073 (2006).

3 Armen, R. S., Uitto, O. D. & Feller, S. E. Phospholipid Component Volumes: Determination and Application to Bilayer Structure Calculations. *Biophysical journal* **75**, 734-744, doi:https://doi.org/10.1016/S0006-3495(98)77563-0 (1998).

4 Gasteiger, E. *et al.* in *The Proteomics Protocols Handbook* (ed John M. Walker) 571-607 (Humana Press, 2005).

5 The UniProt Consortium. UniProt: the universal protein knowledgebase. *Nucleic Acids Research* **45**, D158-D169, doi:10.1093/nar/gkw1099 (2017).

6 Myatt, D. *Biomolecular Scattering Length Density Calculator.* , <http://psldc.isis.rl.ac.uk/Psldc/index.html.> (
